# Causal Relationships Between Total Physical Activity and Ankylosing Spondylitis: A Mendelian Randomization Study

**DOI:** 10.3389/fimmu.2022.887326

**Published:** 2022-07-05

**Authors:** Shaojun Hu, Hongyuan Xing, Xingchen Wang, Ning Zhang, Qiang Xu

**Affiliations:** ^1^ Department of Orthopaedics, 2nd Affiliated Hospital, School of Medicine, Zhejiang University, Hangzhou, China; ^2^ Key Laboratory of Cardiovascular Intervention and Regenerative Medicine of Zhejiang Province, Department of Cardiology, Sir Run Run Shaw Hospital, School of Medicine, Zhejiang University, Hangzhou, China; ^3^ Department of Orthopaedics, Xuzhou Central Hospital, Xuzhou, China

**Keywords:** mendelian randomization, physical activity, ankylosing spondylitis, causal relationship, single-nucleotide polymorphisms

## Abstract

**Background:**

Currently, there is little literature about the association between physical activity (PA) and the risk of ankylosing spondylitis (AS). The present study aimed to understand the causal relationships between PA and AS.

**Methods:**

We performed two-sample Mendelian randomization (MR) using publicly released genome-wide association studies summary statistics to estimate the causal associations of PA with AS risk. The inverse variance weighted (IVW) method was utilized as primary MR analysis. Furthermore, sensitivity, pleiotropy, and heterogeneity analyses were then conducted to assess the robustness of the findings of the present study.

**Results:**

Results of the IVW analysis suggested a protective relationship between accelerometer-based PA and AS (average acceleration, odds ratio [OR] = 0.9995, 95% CI, 0.9988–0.9999, P = 0.014). On the contrary, there was no causal relationship between accelerometer-based PA (acceleration fraction >425 mg; OR = 0.9981, 95% CI = 0.9936–1.0026, P = 0.402) and AS. Furthermore, there was no significant relationship between self-reported vigorous PA and AS (OR = 1.0005, 95% CI = 0.9875–1.0136, P = 0.943), or even between self-reported moderate-to-vigorous PA and AS (OR = 1.0000, 95% CI, 0.9947–1.0052; P = 0.990).

**Conclusions:**

The use of genetic approach in the present study revealed that total physical activity (TPA) has a protective relationship with AS risk. Furthermore, it was evident that vigorous PA or moderate-to-vigorous physical levels are not causally associated with AS. Therefore, the present study evidently supports the hypothesis that enhancing TPA rather than PA intensity is an effective prevention strategy for AS.

## Introduction

Ankylosing spondylitis (AS) is an immune-mediated inflammatory arthritis. It has a strong genetic predisposition to affect the axial skeleton and causes characteristic inflammatory back pain, leading to structural and functional impairment and a reduced quality of life (1, 2). According to different studies conducted in different continents, the annual incidence of AS has ranged from 7 to 32 per 100,000 people (3). A cross-sectional, population-based study hypothesized that the prevalence of AS in Europe was 0.26% (4).

Eighty percent of patients with of AS present the first symptoms under the age of 30 years (5). Furthermore, it has been reported that people in their 30s or 40s are at the highest risk of diagnosing AS, with common symptoms appearing in the lower back and buttocks, as well as frequently in non-specific back conditions. Therefore, diagnosis is often delayed to between 5 and 10 years (6). Currently, the cause of AS is still not known and hence there is no way to prevent AS.

Physical activity (PA) is the mechanical movement produced by skeletal muscle that results in energy expenditure above basal levels and includes exercise, sports, and PAs performed for daily life, work, and leisure (7). It has been reported that long-term adherence to an exercise program can be very beneficial in treatment of AS (8). Furthermore, PA is also promoted as an integral part of standard care throughout the course of spondyloarthritis (7). Precious meta-analysis have shown that home-based PA can efficiently improve disease control and alleviate symptoms of AS (9). However, the role of PA that plays in the etiology of AS has not been well elucidated.

Furthermore, observational studies are susceptible to various biases such as measurement error, potential confounding such as PA improves fitness, physical function, and hence may decrease other risk that affect human health (10), reverse causality. For instance, a previous study have shown that patients with AS have lower levels of PA than normal individuals (11) and even genetic pleiotropy such as PA and rheumatic disease may share a common genetic basis (12). The real causal association between PA and risk of AS may be influenced by potential biases in conventional epidemiology.

Mendelian randomization (MR), which uses instrumental variables (IVs) in the analysis of genetic variants to determine whether there exists an observational association between exposures and outcomes as well as being consistent with a causal effect, has its great advantage (13). First, large amounts of data from genome-wide association studies (GWASs) have been published with very large numbers of IVs and this increases the genetic interpretation of exposure by IVs and is more conducive to accurate as well as reliable results (14). Second, using associations established in observational studies for two cohort studies is equivalent to expanding the sample size of a study and eliminating the bias factors to increase the validity of the test. Third, the current study avoided potential confounding factors because genetic variants were assigned randomly (15). In addition, the risk of reverse causality was minimized because single-nucleotide polymorphism (SNP) alleles were assigned prior to the onset of meiosis. Furthermore, the assumption that total PA (TPA) prevents AS was verified in the present study and the causal effect of different kinds of PA on AS was also estimated through the MR method.

## Methods

### Data Sources

The summary statistics for PA were derived from the recently published GWAS, which was conducted in the UK Biobank cohort (16). The UK Biobank is a large prospective cohort study including nearly 400,000 participants aged between 40 and 69 years (17). Four PA phenotypes were selected from the GWAS, including accelerometer-based PA (average acceleration), accelerometer-based PA (acceleration fraction >425 mg), self-reported moderate-to-vigorous PA, and self-reported vigorous PA). Accelerometer-based PA data (Axivity AX3 wrist-worn accelerometer) were collected from approximately 100,000 study participants (18) whereby the participants were asked to wear a wrist-worn accelerometer at all times for at least 72 h. The analysis was adjusted for assessment center, genotyping array, age, and season.

The present study used genetic variation representing two accelerometer-based PA measures: average acceleration (average acceleration milligrams) and acceleration fraction >425 mg (16). We use instrument-based accelerometer measurements of PA to represent the total amount of exercise. The cutoff value of 425 mg was chosen because it corresponds to an equivalent level of vigorous PA (19). Compared to average acceleration, the leisure PA, whose acceleration fraction <425 mg, is excluded.

For self-reported PA, a touch questionnaire like the International Physical Activity Questionnaire was used on 377,234 participants involved in the UK Biobank to get the data. This method counts the time spent participating in different exercises and classifies them into moderate-intensity activities and vigorous-intensity activities (20).

Genetic associations with AS were derived from Neale Lab Consortium".We find this description is more in line with the norm, including 337,159 British individuals (968 cases and 336,191 controls) imputed using HRC imputation reference panel and adjusted for age, sex, and the first 20 principal components (21).

### Study Design

**Figure 1:**
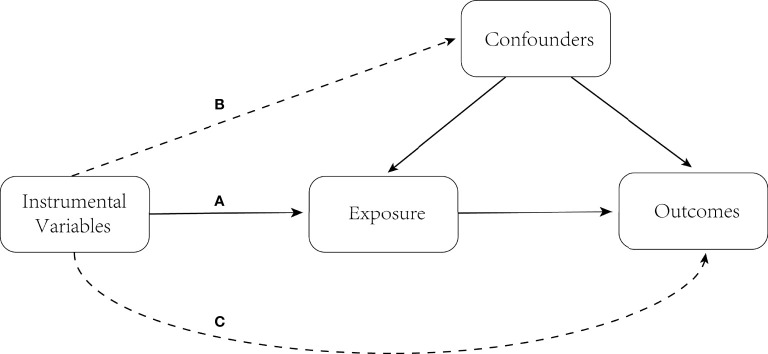
Three key assumptions of MR study. **(A)** The instrumental variables must be associated with exposure (PA); **(B)** instrumental variables must be independent of confounders; and **(C)** instrumental variables should not be directly associated with outcomes (AS). MR, Mendelian randomization; PA, physical activity; AS, ankylosing spondylitis.

In the MR analysis of the current study, the genetic variants that were included as IVs satisfied the following three assumptions (**Figure 1**): (a) IVs must be strongly associated with the exposure (PA); (b) IVs should be independent of any known confounders; (c) SNPs must only be associated with the risk of outcomes (AS) through exposures (PA). Satisfaction of the second and third assumptions in the present study served as a definition of independence from pleiotropy.

### Selection of Instrumental Variables

Initially, the current study extracted SNPs associated with PA at the genome-wide significance threshold [P < 5 × 10^−8^ for average acceleration and self-reported PA, P < 5 × 10^−7^ for PA (acceleration fraction >425 mg)] (21, 22). The present study clumped SNPs using the PLINK clumping algorithm (r^2^ = 0.01 and clumping distance = 5,000 kb). When r^2^ > 0.01 or clumping distance <5,000, the SNPs were removed from the current analysis. This was performed to ensure that each selected SNP was independent without linkage disequilibrium correlation.

The selected IVs should be conditionally independent of AS, and PA levels, and also independent of any known confounders. This ensures that the IVs influence AS only through PA rather than another factors. Therefore, each instrument SNP and its proxies (r² > 0.8) in the PhenoScanncer GWAS database (http://phenoscanner.medschl.cam.ac.uk) were looked up in the current study (18) to assess whether previous SNPs have associations (p < 5 × 10^−8^) with potential related confounders. Furthermore, body mass index (BMI), smoking (23), and diabetes were considered as the major confounders in the present analysis (24–26). After removing the SNPs (**Supplementary Table 1**), 5, 7, 15, and 5 SNPs were used as IVs for TPA, vigorous PA, self-reported moderate-to-vigorous PA, and self-reported vigorous PA, respectively, in the primary analysis.

### MR Analysis

In the main analysis of the current study, the fixed-effects inverse variance-weighted (IVW) method was employed to evaluate the causal effect of PA levels on the risk of AS. A causal estimate was generated for each SNP using the Wald estimator and a corresponding standard error using the Delta method (**Supplementary Table 2**). Subsequently, the fixed-effects IVW method was used to obtain the overall estimate by meta-analyzing all the estimates. It has been reported that, if both pleiotropy and heterogeneity analyses are negative, IVW analysis is the most accurate method in MR (27). Finally, all analyses were carried out using the ‘‘TwoSampleMR’’ package in the R software environment (version 4.1.2).

### Pleiotropy Assessment

The MR-Egger regression was used in the present study to evaluate the horizontal pleiotropic pathway between IVs and AS, independent of PA (28). The MR-Egger regression is an effective way to examine the known bias in meta-analysis which was developed from Egger regression. This approach is expressed as α_i_ = β_γi_+β_0_. In this equation, α_i_ represents the effect between IVs and AS; γi represents the estimated effect between IVs and PA; slope β represents the estimated causal effect of PA on AS; and intercept β_0_ represents the estimated average value of the horizontal pleiotropic pathway.

Furthermore, if intercept β_0_ has p > 0.05, then there was no horizontal pleiotropic pathway exists. Moreover, the slope also gave the estimated pleiotropy-corrected causal effect. The MR-PRESSO test can detect possible outliers, provide adjusted results after excluding the outliers, and thus correct for the horizontal pleiotropy.

### Sensitivity Analysis

Whether the influence of a single SNP disproportionately affected the association was determined by the leave-one-out sensitivity analysis. The MR analysis was performed again leaving out each SNP in turn, and then the overall analysis including all SNPs was also performed and was shown for comparison (29). F-Statistics of all SNPs, which were above the threshold of 10, mean the sensitivity of the result.

### Heterogeneity Assessment

The Cochran’s Q statistic was adopted in the present study to evaluate the between-SNPs heterogeneity between the SNPs in IVW estimates. Funnel and scatter plots of the MR analyses were used to visually assess horizontal pleiotropy and heterogeneity.

## Results

**Figure 2:**
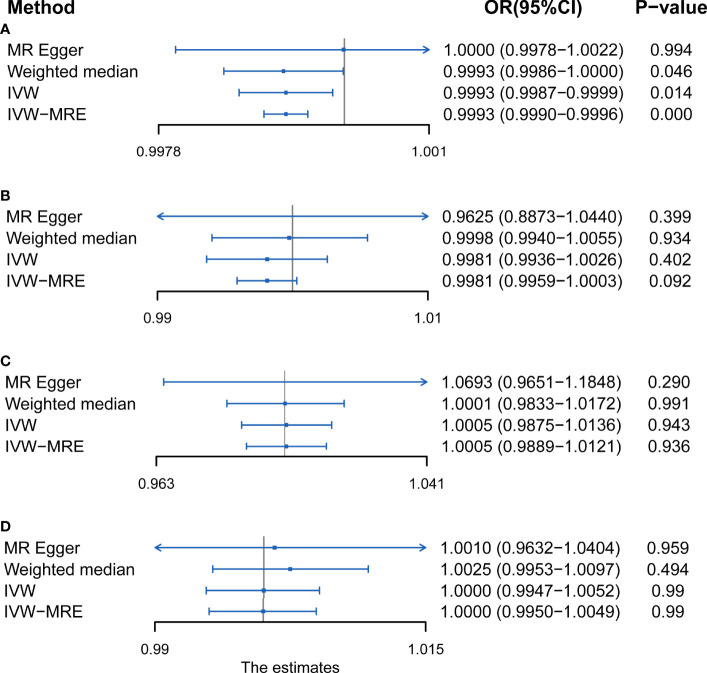
MR results of the association between **(A)** accelerometer-based physical activity (PA) (average acceleration), **(B)** accelerometer-based PA (acceleration fraction >425 mg), **(C)** self-reported vigorous PA, and **(D)** self-reported moderate-to-vigorous PA with AS risk. OR, odds ratio; CI, confidence interval; IVW, inverse variance weighted; IVW-MRE, inverse variance weighted (multiplicative random effects).

Results of the present study showed that the F-statistics of all SNPs were above the threshold of 10 (**Supplementary Table S3**). This implies that the weak instrument bias was absent and also the sensitivity of the analysis. In addition, the leave-one-out associations approach also confirmed that the result of the current study is sensitive (**Supplementary Figure 1**). The p-value for the MR-Egger intercept test and MR-PRESSO test (**Supplementary Table S4**) as well as the symmetry in funnel plots (**Supplementary Figure 2**) of the present study demonstrated the absence of horizontal pleiotropy. The Cochran’s Q statistic test (**Supplementary Table S5**), funnel plots (**Supplementary Figure 2**), and scatter plots demonstrated that there is no heterogeneity in the results of the present study. Statistical methods of IVW, IVW of multiplicative random effects, weighted median, and MR-Egger were used to draw conclusions about the clear relationship between PA and AS (**Figure 2**).

### Accelerometer- Based PA and AS

**Figure 3:**
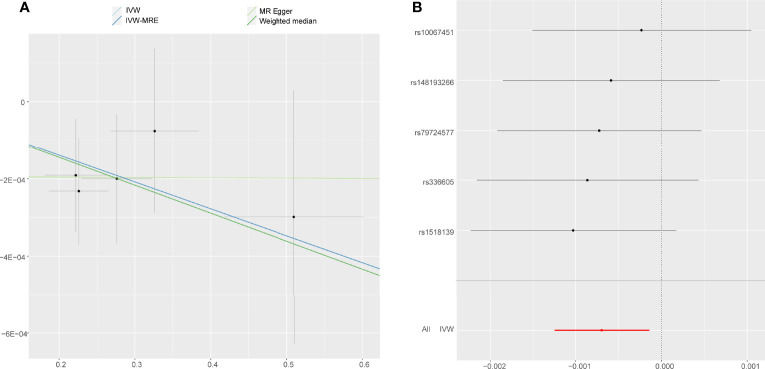
Forest plot and scatter plot of the association of accelerometer-based PA (average acceleration) with the risk of AS. **(A)** Scatter plot: each black dot indicates a SNP, plotted by the estimate of SNP on accelerometer-based PA (average acceleration) and the estimate of SNP on the risk of AS with standard error bars. The slopes of the lines correspond to causal estimates using each of the different methods. **(B)** Forest plot: the dot and bar indicate the causal estimate of accelerometer-based PA (average acceleration) on risk of AS.

Using IVW examine, an evidence of a protective causal relationship between accelerometer-based PA (average acceleration) with AS was found in the findings of the present study [OR, 0.9995 for AS per 1-SD increase in mean acceleration; 95% confidence interval (CI), 0.9988–0.9999; P = 0.014] (**Figure 3**). Remarkably, similar results were observed upon employing various other statistical methods (**Figure 2A**).

**Figure 4:**
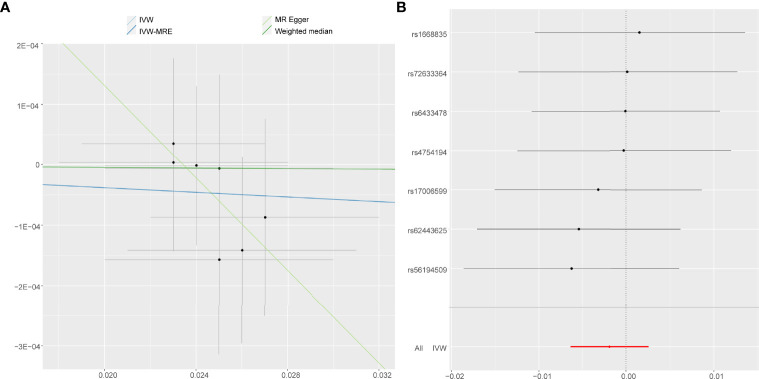
Forest plot and scatter plot of the association of accelerometer-based PA (acceleration fraction >425 mg) with the risk of AS. **(A)** Scatter plot: each black dot indicates a SNP, plotted by the estimate of SNP on accelerometer-based PA (acceleration fraction >425 mg) and the estimate of SNP on the risk of AS with standard error bars. The slopes of the lines correspond to causal estimates using each of the different methods. **(B)** Forest plot: the dot and bar indicate the causal estimate of accelerometer-based PA (acceleration fraction >425 mg) on risk of AS.

On the contrary, it was evident that, regardless of instrument SNP threshold, there was no statistically significant evidence of a relationship between accelerometer-based PA (acceleration fraction >425 mg) and AS (OR, 0.9981 for AS per 1-SD increase in mean acceleration; 95% CI, 0.9936–1.0026; P = 0.402, using IVW) (**Figure 4**). Other statistical methods draw the same conclusion (**Figure 2B**).

### Self-Reported PA and AS

**Figure 5:**
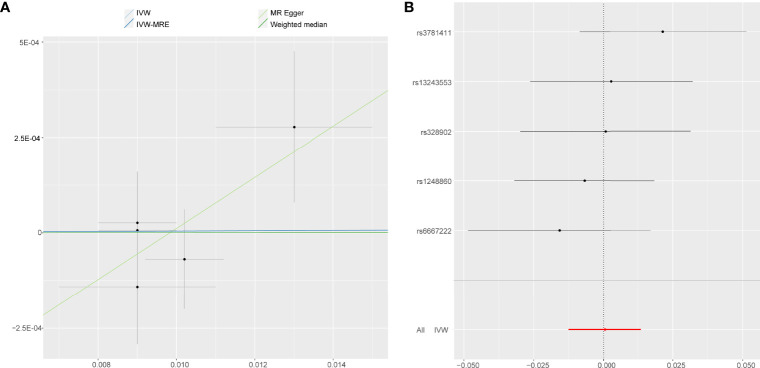
Forest plot and scatter plot of the association of self-reported vigorous PA with the risk of AS. **(A)** Scatter plot: each black dot indicates a SNP, plotted by the estimate of SNP on self-reported vigorous PA and the estimate of SNP on the risk of AS with standard error bars. The slopes of the lines correspond to causal estimates using each of the different methods. **(B)** Forest plot: the dot and bar indicate the causal estimate of self-reported vigorous PA on risk of AS.

**Figure 6:**
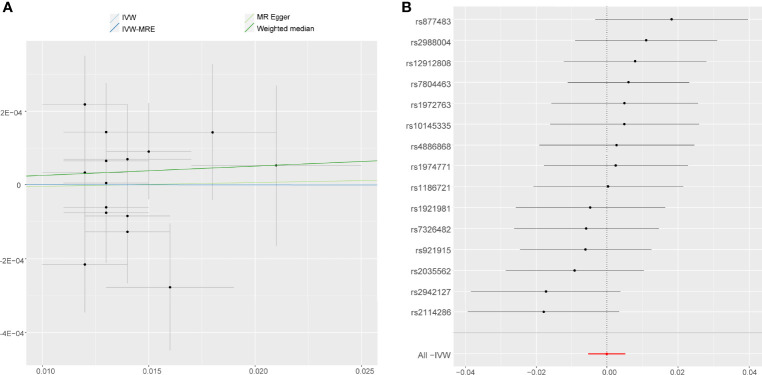
Forest plot and scatter plot of the association of self-reported moderate-to-vigorous PA with the risk of AS. **(A)** Scatter plot: each black dot indicates a SNP, plotted by the estimate of SNP on self-reported moderate-to-vigorous PA and the estimate of SNP on the risk of AS with standard error bars. The slopes of the lines correspond to causal estimates using each of the different methods. **(B)** Forest plot: the dot and bar indicate the causal estimate of self-reported moderate-to-vigorous PA on risk of AS.

Just like accelerometer-based PA (acceleration fraction >425 mg), self-reported vigorous PA and self-reported moderate-to-vigorous PA showed no statistically significant evidence that reducing the risk of developing AS. According to the results obtained on the IVW analysis of the present study, it was evident that the OR and 95% CI per unit increase in self-reported vigorous PA within AS were 1.0005 (0.9875–1.0136), P = 0.943 (**Figure 5**), whereas that of in self-reported moderate-to-vigorous PA within AS were 1.0000 (0.9947–1.0052), P = 0.990 (**Figure 6**). Similar results were observed upon employing various other statistical methods (**Figures 2C, D**).

## Discussion

AS is a chronic and inflammatory disease of the axial spine which can result in severe chronic pain and disability (1). However, although environment and genetics can influence AS, its exact cause of is still unknown. Our study used MR with genetic IVs selected from large-scale GWAS to find evidence supporting a potential causal relationship between TPA and a reduced risk for AS. It was evident that there was no causal relationship between either moderate-to-vigorous or vigorous PA and AS.

Previously reported literature has demonstrated therapeutic benefits of PA for AS, including improving the symptoms of AS (30) and reducing global patient assessment of disease activity (31). Kabul et al. show the manual soft tissue mobilization with spinal mobility exercises that significantly reduced deterioration of the disease condition (32). In addition, PA is advocated for as an integral part of standard care throughout the course of AS (7). AS self-management program, which includes PA, can provide long-term improvement in disease symptoms, reduce disease activity, and significantly improve quality of life (33). Elsewhere, it has been found that high disease activity of AS is related to low levels of PA in patients (34). The study has shown that spinal mobility, aerobic capacity, forced vital capacity, and cardiac autonomic regulation were significantly lower in patients with AS and were associated with disease activity (35–37). Enthesitis is related to deteriorated static foot posture in patients with AS (38). Patients with AS also have lower levels of exercise as compared with normal individuals (39). Therefore, people with AS have reduced levels of PA (40); it is difficult to evaluate whether the level of exercise has an effect on the development of AS.

To avoid this dilemma, the present study used IVs to analyze genetic variation and MR to determine whether there was an observed association and causal effect between exposures (PA) and outcomes (AS). Findings of the present study were in line with previous meta-analysis, which suggest that higher TPA, not including vigorous PA, is associated with AS (9, 41, 42). Furthermore, it has also been suggested that home-based PA can improve disease activity and improve pain function. In addition, it was concluded that an increase in TPA has a protective association with AS. Furthermore, the causal relationship in the current study appeared to be motivated by TPA, but not moderate-to-vigorous PA or vigorous PA, and this meant that the TPA may be more important than the levels of activity.

Self-reported vigorous, self-reported moderate-to-vigorous, and instrument-measured vigorous PAs were all unprotective against AS, which is consistent in the present study. A long-term cross-sectional study showed a significant reduction in motor ability in patients with AS (43). Previously, it has been reported that, regardless of disease activity, the amount of vigorous and moderate exercise in people with ankylosing spine is significantly lower than normal (44). Less exercise may be due to the fact that the disease (AS) can cause stiffness in the joints (45) and reduction in muscle strength of the whole body (46), rather than a decrease in moderate-to-vigorous PA or vigorous PA and hence making the disease (AS) more likely to occur. Usually, higher PA is linked to other healthy lifestyle factors, including lower prevalence of obesity and smoking (47).

Conventional multivariable regression methods may limit the ability to disentangle the impact of highly correlated healthy lifestyle habits from each other and from other positive effects associated with PA and subsequently with a lower risk of AS such as BMI (48). Patients with a screening diagnosis of AS may be able to make lifestyle changes to delay manifestation of the disease. The lifestyle changes may include increasing levels of PA, losing body weight, and quitting smoking. This will bring reverse causality to the study of the relationship between PA and AS, and hence, it is difficult to establish causality.

On the contrary to observational studies of PA and randomized controlled trials, the main strength of the present study was the use of MR, which can reflect the causal factors different from short-term self-reported PA habits or lifetime exposure to PA interventions of shorter duration. Another advantage of the current study was that the MR study design not only reduces unobserved confounding but also avoids the creation of reverse causality. To minimize the possibility of biased results, the current study used a very large sample size (330,000 patients in the AS study and a total of nearly 400,000 patients in the PA study) and this provided potential to detect effect sizes previously reported in observational studies.

Although sensitivity analyses were also conducted to rule out horizontal pleiotropy, it is not possible to completely rule out pleiotropic mechanisms because the biological role of these SNPs was still not fully understood. While horizontal pleiotropy is a concern for MR inference, the vertical pleiotropy irradiation acting on outcomes along the same causal pathway through other variables is acceptable. For instance, if PA leads to a decrease in BMI, then the BMI may in turn affect AS, which would represent vertical pleiotropy and therefore MR estimation should not be unnecessarily penalized. Reassuringly, it was found that the MR estimates observed through sensitivity analysis (leave-one-out associations) in the present study were robust, indicating that bias from apparent sources of pleiotropy was negligible.

However, our study has limitations. Although the data are statistically significant, this does not mean that it is clinically relevant and therefore more clinical trials need to be conducted to verify this finding.

Results of AS may be affected by a degree of misclassification, as the condition-determining studies included in the Neale Lab consortium follow certain norms, AS was not specifically classified (with or without comorbidities and different disease courses among others) and AS was self-reported by the enumerator, which allows for some bias. The self-reported prevalence of AS by the Neale Lab consortium was 0.3%, this was within the European range of AS prevalence (2), has a large sample, and so the outcome which was used in the present study was still plausible. Therefore, it is recommended that a large sample of GWAS data should be used in the future with more definitive diagnosis and stratification of AS for subsequent, more in-depth analysis when it becomes available.

Self-report measures of activity may be affected by mood states and cognitive biases. Although this does not invalidate the utility of self-report measures (49), it is necessary to validate their findings with objective measures. This is because objectively measured PA is more likely to be inherited and therefore may be closer to the biological processes that PA directly influences AS. The advantage of accelerometers for objective monitoring of PA is their ability to quantify dynamic activity during walking and swimming among other activities.

However, accelerometers also have limitations. It is difficult to measure posture and sedentary, light activity, and non-ambulatory activity (cycling) and thus there is a need for further study on the relationship between light PA and AS. In addition, habitual behavior may also change when participants are aware that they are being detected by the instrument, which seems difficult to avoid. Therefore, this experiment used both self-reported PA and accelerometer-measured PA together to examine the effects of vigorous PA on AS and consistently concluded that vigorous PA is not protective against AS. These findings were further in consonance with the conclusion of the present study. However, despite the limitations of accelerometers in the present study, the application of MR provided independent support for a potential protective relationship between TPA and AS risk through genetic instrumentation.

Furthermore, as a tool for causal inference, MR removes the typical challenges of observational studies by suggesting and providing independent support for a potentially protective relationship between TPA and AS. Moreover, it is vital to present a stronger evidence of causality because few modifiable factors are known to prevent AS.

It would be of great interest if TPA did reduce the risk of AS. To a large extent, it has been found that the global variation in prevalence of AS depends on the prevalence of HLA-B27 (3). Furthermore, HLA-B27 has been suggested to be important in pathogenesis of AS and hence contributing to 20% of AS heritability (50). This means that HLA-B27–positive carriers can reduce the incidence of AS by increasing their level of TPA. This allows PA measurement to be more targeted to susceptible people to reduce the risk of AS and can also generate public health returns on levels of human productivity and reduce the burden of healthcare as well as the risk of personal development AS.

## Conclusions

In conclusion, the present study used a genetic approach and evidently found that TPA is causally associated with AS risk. Furthermore, it was evident that there is no protective relationship between vigorous PA or moderate-to-vigorous PA and AS. Therefore, the current study affirmatively supported hypothesis that enhancing TPA rather than PA intensity is an effective prevention strategy for AS.

## Data Availability Statement

The datasets presented in this study can be found in online repositories. The names of the repository/repositories and accession number(s) can be found in the article/**Supplementary Material**.

## Author Contributions

SH conceived the presented idea. SH, HX, and XW performed the computations and manuscript writing. SH and HX were involved in acquisition of data. SH was involved in interpretation of data. NZ and QX contributed to the preparation and supervision of the final version of the manuscript. All authors contributed to the article and approved the submitted version.

## Funding

This work was supported by the National Natural Science Foundation of China (grant No. 81972514).

## Conflict of Interest

The authors declare that the research was conducted in the absence of any commercial or financial relationships that could be construed as a potential conflict of interest.

## Publisher’s Note

All claims expressed in this article are solely those of the authors and do not necessarily represent those of their affiliated organizations, or those of the publisher, the editors and the reviewers. Any product that may be evaluated in this article, or claim that may be made by its manufacturer, is not guaranteed or endorsed by the publisher.
