# Supplementary material for: Causal Relationships Between Total Physical Activity and Ankylosing Spondylitis: A Mendelian Randomization Study
Source: Front Immunol. 2022 Jul 5;13:887326. doi: 10.3389/fimmu.2022.887326 (PMC9294357; doi:10.3389/fimmu.2022.887326)
Supplement: Supplementary file 1 [file DataSheet_1.docx]

**SUPPLEMENTARY MATERIAL**

**Supplementary Figure Legend**

**Supplementary Figure 1. Funnel plots of accelerometer-based PA (average acceleration), accelerometer-based PA (acceleration fraction >425 milligrams), self-reported vigorous PA and self-reported moderate-to-vigorous PA against the genetic associations with ankylosing spondylitis.**

Funnel plot to assess heterogeneity of genetic associations with accelerometer-based PA (average acceleration), accelerometer-based PA (acceleration fraction >425 milligrams), self-reported vigorous PA and self-reported moderate-to-vigorous PA against the genetic associations with ankylosing spondylitis. The light blue line represents the inverse-variance weighted estimate, and the dark blue line represents the MR‐Egger estimate. (A: accelerometer-based PA (average acceleration); B: accelerometer-based PA (acceleration fraction >425 milligrams); C: self-reported vigorous PA; D: self-reported moderate-to-vigorous PA)

PA: physical activity

**Supplementary Figure 2. Leave-one-out sensitivity analysis of single SNP of accelerometer-based PA (average acceleration), accelerometer-based PA (acceleration fraction >425 milligrams), self-reported vigorous PA and self-reported moderate-to-vigorous PA for ankylosing spondylitis.**

Leave-one-out sensitivity analysis is performed to ascertain if an association is being disproportionately influenced by a single SNP for ankylosing spondylitis (A: accelerometer-based PA (average acceleration); B: accelerometer-based PA (acceleration fraction >425 milligrams); C: self-reported vigorous PA; D: self-reported moderate-to-vigorous PA). Each black point in the forest plot represents the MR analysis (using IVW) excluding that particular SNP. The overall analysis including all SNPs is also shown for comparison.

SNP: Single Nucleotide Polymorphism

**Supplementary Table 1: Deleted SNPs associated with confounders**

| SNP | Reason for deletion |
| --- | --- |
| rs743580 | BMI |
| rs429358 | BMI, type 2 diabetes |
| rs9276758 | Type 1 diabetes |
| rs34517439 | BMI |
| rs7084454 | BMI |

SNP: Single Nucleotide Polymorphism; BMI: body mass index

**Supplementary Table 2:MR analysis results of a single SNP.**

| **Traits** | **Beta** | **SE** | **P-value** |
| --- | --- | --- | --- |
| **Average accelerometer-based PA** |  |  |  |
| rs10067451 | -2.32E-04 | 6.52E-04 | 0.723 |
| rs148193266 | -5.86E-04 | 6.46E-04 | 0.364 |
| rs1518139 | -1.03E-03 | 6.13E-04 | 0.094 |
| rs336605 | -8.61E-04 | 6.60E-04 | 0.192 |
| rs79724577 | -7.24E-04 | 6.09E-04 | 0.234 |
| All - Inverse variance weighted | -6.95E-04 | 2.84E-04 | 0.014 |
| All - MR Egger | -9.20E-06 | 1.11E-03 | 0.994 |
| **Average accelerometer-based PA**  **(fraction of accelerations >425 milligravities)** |  |  |  |
| rs1668835 | 1.53E-03 | 6.13E-03 | 0.803 |
| rs17006599 | -3.23E-03 | 6.04E-03 | 0.593 |
| rs4754194 | -2.59E-04 | 6.23E-03 | 0.967 |
| rs56194509 | -6.28E-03 | 6.28E-03 | 0.318 |
| rs62443625 | -5.44E-03 | 5.94E-03 | 0.359 |
| rs6433478 | -6.82E-05 | 5.49E-03 | 0.990 |
| rs72633364 | 1.48E-04 | 6.38E-03 | 0.981 |
| All - Inverse variance weighted | -1.92E-03 | 2.29E-03 | 0.402 |
| All - MR Egger | -3.83E-02 | 4.15E-02 | 0.399 |
| **Self-reported vigorous PA** |  |  |  |
| rs1248860 | -6.76E-03 | 1.28E-02 | 0.598 |
| rs13243553 | 2.93E-03 | 1.49E-02 | 0.844 |
| rs328902 | 7.46E-04 | 1.56E-02 | 0.962 |
| rs3781411 | 2.14E-02 | 1.53E-02 | 0.161 |
| rs6667222 | -1.57E-02 | 1.67E-02 | 0.347 |
| All - Inverse variance weighted | 4.75E-04 | 6.65E-03 | 0.943 |
| All - MR Egger | 6.70E-02 | 5.23E-02 | 0.290 |
| **Self-reported moderate-to-vigorous PA** |  |  |  |
| rs10145335 | 4.91E-03 | 1.07E-02 | 0.647 |
| rs1186721 | 3.41E-04 | 1.08E-02 | 0.975 |
| rs12912808 | 7.94E-03 | 1.02E-02 | 0.438 |
| rs1921981 | -4.70E-03 | 1.08E-02 | 0.662 |
| rs1972763 | 4.94E-03 | 1.06E-02 | 0.641 |
| rs1974771 | 2.48E-03 | 1.04E-02 | 0.811 |
| rs2035562 | -9.08E-03 | 9.95E-03 | 0.362 |
| rs2114286 | -1.79E-02 | 1.09E-02 | 0.101 |
| rs2942127 | -1.73E-02 | 1.08E-02 | 0.108 |
| rs2988004 | 1.11E-02 | 1.02E-02 | 0.278 |
| rs4886868 | 2.71E-03 | 1.12E-02 | 0.808 |
| rs7326482 | -5.82E-03 | 1.04E-02 | 0.577 |
| rs7804463 | 6.07E-03 | 8.72E-03 | 0.486 |
| rs877483 | 1.82E-02 | 1.10E-02 | 0.097 |
| rs921915 | -6.03E-03 | 9.47E-03 | 0.525 |
| All - Inverse variance weighted | -3.23E-05 | 2.67E-03 | 0.990 |
| All - MR Egger | 1.04E-03 | 1.97E-02 | 0.959 |

Beta: beta coefficient; SE: standard error; PA: physical activity

**Supplementary Table 3: Characteristics of the SNPs used as instrumental variables in the MR analysis (1,2)**

| **SNP** | **Chr** | **Position** | **EA** | **OA** | **EAF** | **PA** | | | **AS** | | | **F** |
| --- | --- | --- | --- | --- | --- | --- | --- | --- | --- | --- | --- | --- |
|  |  |  |  |  |  | **Beta** | **SE** | **P-value** | **Beta** | **SE** | **P-value** |  |
| Average accelerometer-based PA | | | | | | | | | | | | |
| rs336605 | 3 | 18614858 | G | T | 0.276 | 0.222 | 0.041 | 4.50E-08 | -1.91E-04 | 1.47E-04 | 0.19 | 30 |
| rs10067451 | 5 | 88646688 | G | A | 0.887 | 0.326 | 0.058 | 2.00E-08 | -7.55E-05 | 2.13E-04 | 0.72 | 31 |
| rs148193266 | 11 | 104657953 | A | C | 0.957 | -0.51 | 0.092 | 3.10E-08 | 2.99E-04 | 3.29E-04 | 0.36 | 31 |
| rs79724577 | 17 | 45386127 | A | C | 0.818 | -0.276 | 0.047 | 4.60E-09 | 2.00E-04 | 1.68E-04 | 0.23 | 34 |
| rs1518139 | 18 | 43171267 | G | T | 0.662 | -0.226 | 0.039 | 4.50E-09 | 2.32E-04 | 1.38E-04 | 0.09 | 34 |
| Average accelerometer-based PA (fraction of accelerations >425 milligravities) | | | | | | | | | | | | |
| rs17006599 | 1 | 219763423 | A | G | 0.8 | 0.027 | 0.005 | 1.00E-07 | -8.71E-05 | 1.63E-04 | 0.59 | 28 |
| rs6433478 | 2 | 174376754 | T | C | 0.457 | -0.024 | 0.004 | 1.00E-08 | 1.64E-06 | 1.32E-04 | 0.99 | 32 |
| rs62443625 | 7 | 39013531 | T | C | 0.767 | -0.026 | 0.005 | 1.00E-07 | 1.41E-04 | 1.54E-04 | 0.36 | 28 |
| rs72633364 | 8 | 34329370 | G | A | 0.711 | -0.023 | 0.005 | 4.10E-07 | -3.41E-06 | 1.47E-04 | 0.98 | 26 |
| rs4754194 | 11 | 107219461 | C | T | 0.773 | -0.025 | 0.005 | 2.40E-07 | 6.47E-06 | 1.56E-04 | 0.97 | 27 |
| rs56194509 | 17 | 45767193 | T | G | 0.78 | -0.025 | 0.005 | 3.90E-07 | 1.57E-04 | 1.57E-04 | 0.32 | 26 |
| rs1668835 | 18 | 24898988 | T | A | 0.688 | -0.023 | 0.004 | 3.10E-07 | -3.52E-05 | 1.41E-04 | 0.80 | 26 |
| Self-reported moderate-to-vigorous PA | | | | | | | | | | | | |
| rs10145335 | 14 | 98547748 | G | A | 0.75 | -0.014 | 0.003 | 2.70E-08 | -6.87E-05 | 1.50E-04 | 0.65 | 22 |
| rs1186721 | 7 | 34974602 | G | A | 0.68 | -0.013 | 0.002 | 4.40E-08 | -4.43E-06 | 1.40E-04 | 0.97 | 42 |
| rs12912808 | 15 | 95292223 | C | T | 0.85 | 0.018 | 0.003 | 1.70E-08 | 1.43E-04 | 1.84E-04 | 0.44 | 36 |
| rs1972763 | 4 | 159860563 | C | T | 0.34 | 0.013 | 0.002 | 3.30E-08 | 6.42E-05 | 1.38E-04 | 0.64 | 42 |
| rs1921981 | 21 | 42422547 | G | A | 0.67 | 0.013 | 0.002 | 3.80E-08 | -6.11E-05 | 1.40E-04 | 0.66 | 42 |
| rs1974771 | 2 | 54278543 | G | A | 0.9 | -0.021 | 0.004 | 6.60E-09 | -5.20E-05 | 2.18E-04 | 0.81 | 28 |
| rs2035562 | 3 | 85056521 | A | G | 0.33 | -0.014 | 0.002 | 3.90E-09 | 1.27E-04 | 1.39E-04 | 0.36 | 49 |
| rs2114286 | 3 | 41194283 | A | G | 0.47 | -0.012 | 0.002 | 3.30E-08 | 2.15E-04 | 1.31E-04 | 0.10 | 36 |
| rs2942127 | 1 | 204420067 | G | A | 0.18 | 0.016 | 0.003 | 3.30E-08 | -2.77E-04 | 1.72E-04 | 0.11 | 28 |
| rs2988004 | 9 | 37044388 | T | G | 0.56 | -0.013 | 0.002 | 4.10E-09 | -1.44E-04 | 1.33E-04 | 0.28 | 42 |
| rs4886868 | 15 | 74353561 | T | G | 0.41 | -0.012 | 0.002 | 3.50E-08 | -3.26E-05 | 1.34E-04 | 0.81 | 36 |
| rs7326482 | 13 | 54037803 | G | T | 0.38 | -0.013 | 0.002 | 1.60E-08 | 7.57E-05 | 1.36E-04 | 0.58 | 42 |
| rs7804463 | 7 | 133447651 | T | C | 0.53 | 0.015 | 0.002 | 1.20E-11 | 9.11E-05 | 1.31E-04 | 0.49 | 56 |
| rs877483 | 3 | 53846741 | T | C | 0.43 | 0.012 | 0.002 | 4.00E-08 | 2.18E-04 | 1.32E-04 | 0.10 | 36 |
| rs921915 | 7 | 50228581 | T | C | 0.41 | -0.014 | 0.002 | 5.70E-10 | 8.44E-05 | 1.33E-04 | 0.52 | 49 |
| Self-reported vigorous PA | | | | | | | | | | | | |
| rs1248860 | 3 | 85015779 | G | A | 0.48 | -0.0102 | 0.001 | 1.10E-13 | 6.90E-05 | 1.31E-04 | 0.60 | 104 |
| rs13243553 | 7 | 133506955 | G | A | 0.61 | 0.009 | 0.001 | 9.00E-11 | 2.64E-05 | 1.34E-04 | 0.84 | 81 |
| rs328902 | 7 | 35020843 | C | T | 0.69 | -0.009 | 0.001 | 5.50E-10 | -6.71E-06 | 1.40E-04 | 0.96 | 81 |
| rs3781411 | 10 | 126715436 | C | T | 0.88 | 0.013 | 0.002 | 3.00E-10 | 2.78E-04 | 1.98E-04 | 0.16 | 42 |
| rs6667222 | 1 | 154253661 | A | C | 0.75 | 0.009 | 0.002 | 8.70E-09 | -1.41E-04 | 1.50E-04 | 0.35 | 20 |

SNP: Single Nucleotide Polymorphism; Chr: chromosome; EA: Effect Allele; OA: Non‐Effect Allele; EAF: Effect allele frequency; Beta: beta coefficient; SE: standard error.

**Supplementary Table 4: horizontal pleiotropy analysis of accelerometer-based PA (average acceleration), accelerometer-based PA (acceleration fraction >425 milligrams), self-reported vigorous PA and self-reported moderate-to-vigorous PA for ankylosing spondylitis**

| Exposure | Egger intercept | SE | P-value | MR-PRESSO test |
| --- | --- | --- | --- | --- |
| Self-reported vigorous PA | -0.00065797 | 0.000513 | 0.29 | NA |
| Average accelerometer-based PA (fraction of accelerations >425 milligravities) | 0.00089589 | 0.001022 | 0.42 | NA |
| Self-reported moderate-to-vigorous PA | -1.49E-05 | 0.000272 | 0.95 | NA |
| Average accelerometer-based PA | -0.000194061 | 0.000305 | 0.56 | NA |

SE: standard error; PA: physical activity

**Supplementary Table 5:** **heterogeneity analysis and MR-PRESSO analysis of accelerometer-based PA (average acceleration), accelerometer-based PA (acceleration fraction >425 milligrams), self-reported vigorous PA and self-reported moderate-to-vigorous PA for ankylosing.**

| **Outcome** | **Exposure** | **Method** | **Q** | **Q_df** | **P-value for heterogeneity** |
| --- | --- | --- | --- | --- | --- |
| Non-cancer illness code self-reported: ankylosing spondylitis \|\| id:ukb-a-88 | Self-reported moderate-to-vigorous PA | MR Egger | 12.57865 | 13 | 0.48 |
|  |  | IVW | 12.58166 | 14 | 0.55 |
|  |  | IVW-MRE | 12.58166 | 14 | 0.55 |
|  | Self-reported vigorous PA | MR Egger | 1.520872 | 3 | 0.67 |
|  |  | IVW | 3.163097 | 4 | 0.53 |
|  |  | IVW-MRE | 3.163097 | 4 | 0.53 |
|  | Average accelerometer-based PA | MR Egger | 0.487999 | 3 | 0.92 |
|  |  | IVW | 0.892617 | 4 | 0.92 |
|  |  | IVW-MRE | 0.892617 | 4 | 0.92 |
|  | Average accelerometer-based PA (fraction of accelerations >425) | MR Egger | 0.717447 | 5 | 0.98 |
|  |  | IVW | 1.486301 | 6 | 0.96 |
|  |  | IVW-MRE | 1.486301 | 6 | 0.96 |

PA: physical activity; IVW: Inverse variance weighted; IVW-MRE: Inverse variance weighted (multiplicative random effects)

**Reference**

1. Meisinger C, Linseisen J, Leitzmann M, Baurecht H, Baumeister SE. Association of physical activity and sedentary behavior with type 2 diabetes and glycemic traits: a two-sample Mendelian randomization study. BMJ Open Diabetes Res Care. 2020 Dec 8;8(2):e001896.

2. Sun L, Zhu J, Ling Y, Mi S, Li Y, Wang T, et al. Physical activity and the risk of rheumatoid arthritis: evidence from meta-analysis and Mendelian randomization. International Journal of Epidemiology. 2021 Nov 10;50(5):1593–603.
