# Supplementary figures and images for: Causal Relationships Between Total Physical Activity and Ankylosing Spondylitis: A Mendelian Randomization Study
Source: Front Immunol. 2022 Jul 5;13:887326. doi: 10.3389/fimmu.2022.887326 (PMC9294357; doi:10.3389/fimmu.2022.887326)

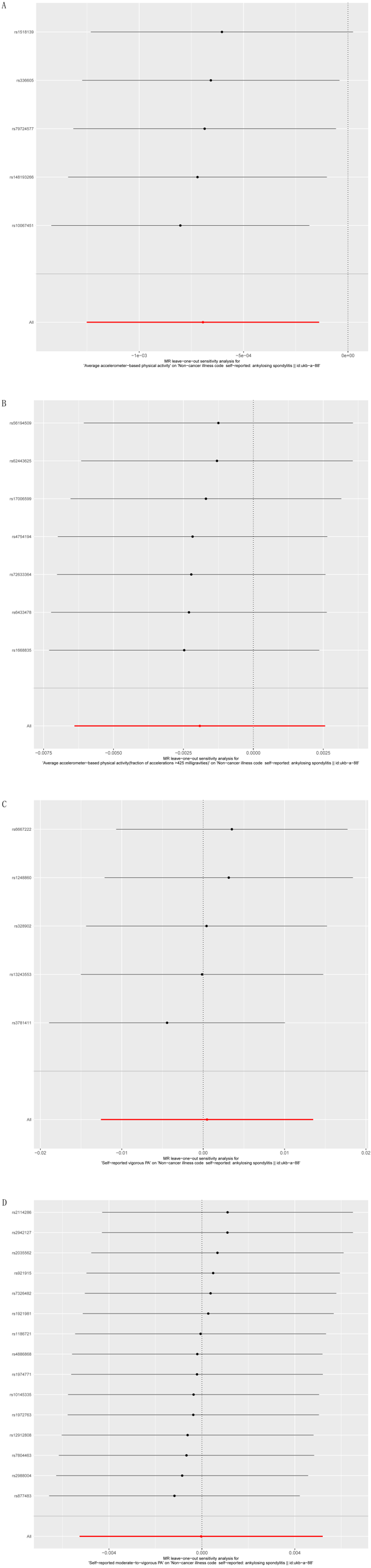

Supplement: Supplementary file 2 [file Image_1.tif]

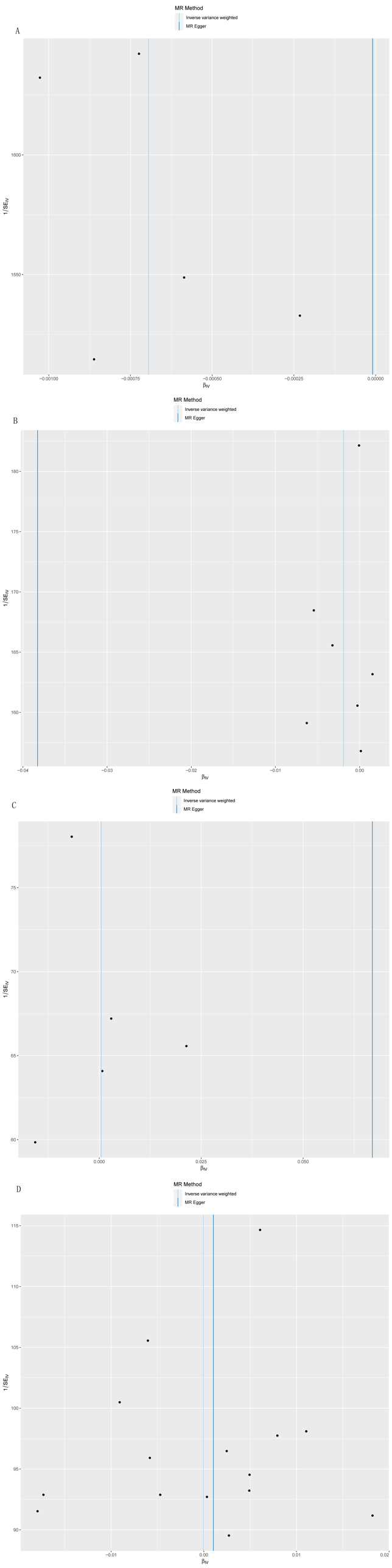

Supplement: Supplementary file 3 [file Image_2.tif]
